# Chip scale coil stabilized Brillouin laser driving a room temperature trapped ion qubit

**DOI:** 10.1038/s41467-026-69948-2

**Published:** 2026-03-03

**Authors:** Nitesh Chauhan, Christopher Caron, Andrei Isichenko, Meiting Song, Zhenyu Wei, Nishat Helaly, Kaikai Liu, Jiawei Wang, Robert J. Niffenegger, Daniel J. Blumenthal

**Affiliations:** 1 Department of Electrical and Computer Engineering, University of California Santa Barbara, Santa Barbara, CA USA; 2 Department of Electrical and Computer Engineering, University of Massachusetts Amherst, Amherst, MA USA

**Keywords:** Lasers, LEDs and light sources, Atom optics, Quantum optics

## Abstract

Photonic integrated stable, ultra-low-noise lasers are essential for scalable and portable quantum information systems. Trapped ions are a leading modality for quantum computing and optical clocks, with room-temperature operation enabling portable applications. Current systems rely on free-space lasers and stabilization cavities, frequency conversion, and cryogenic infrastructure, limiting size, weight, and power. We demonstrate a chip-scale coil-stabilized 674 nm Brillouin laser driving qubit state preparation and measurement and the optical clock transition in a room-temperature surface electrode trapped ^88^Sr^+^ ion without a bulk-optic reference cavity. The CMOS compatible silicon nitride integrated 3-meter coil and Brillouin laser achieve 8.8×10^-13^ stability at 20 ms, sufficient to interrogate the 0.4 Hz quadrupole optical clock transition. The ion-disciplined laser achieves 5.3 $\times {10}^{-13}/\sqrt{\tau }$ stability, spectroscopy with 1.5 kHz linewidths, and 99.6% qubit state preparation and measurement fidelity. These results light the way towards integration of stabilized lasers with trapped-ion chips for portable and robust quantum technologies.

## Abstract (web summary)

Stable, ultra-low-noise integrated lasers are essential for scalable quantum computers and portable optical clocks. Here, authors demonstrate a chip-scale coil stabilized Brillion laser driving a room-temperature trapped-ion clock and qubit without a bulk reference cavity achieving 99.6% SPAM fidelity.

## Introduction

Atomic, ion, and molecular quantum experiments form a fundamental technology platform for quantum information sciences, enabling quantum information processors^1^, optical clocks^2^, precision quantum-logic spectroscopy^3^, and quantum sensors^4^. In particular, ion-based qubit experiments employ a physics package that contains the ion trap in addition to laser and optical infrastructure used to trap and cool the ion, interrogate the clock transition, and perform qubit state preparation and measurement (SPAM)^5^. Today, this laser and optical infrastructure occupies table tops and racks of equipment supporting functions including light generation and stabilization, modulation, detection, control, and beam delivery to the ion using optical fiber and free space optics. The volume and complexity of this infrastructure adds to the size, weight, and cost of the overall system, limits reliability and scalability, and introduces laser phase instability at the ion that must be mitigated. Integration of the lasers and optical infrastructure can provide compact, scalable solutions for trapped-ion qubit systems as well as have broad impact to areas including quantum sensing, portable precision timekeeping and metrology, and space-based quantum experiments.

**Fig. 1:**
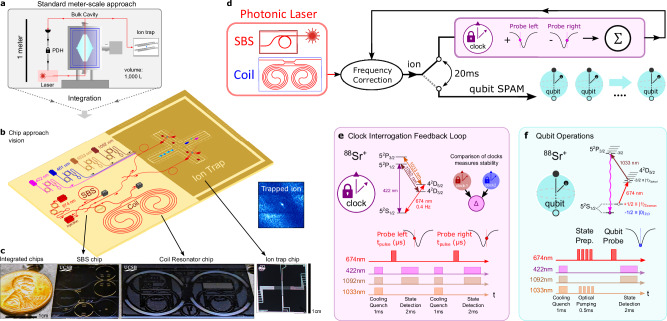
Chip-scale ultra-narrow linewidth stabilized Brillouin laser for trapped ion qubit operations. **a** Conventional optical qubits utilize stabilized lasers consisting of meter-scale ECDLs and vacuum isolated bulk cavities for interrogating optical clock transitions. **b** Vision for Si_3_N_4_ photonic integration of lasers, photonics, and SET including the chip-scale stabilized laser. All lasers, modulators, photodetectors, and other components can be integrated on the same Si_3_N_4_ platform^32,34,35,55^. **c** From left to right, photograph of penny (for scale), Si_3_N_4_ integrated SBS laser, Si_3_N_4_ integrated 3-meter coil-resonator^32^, and integrated ion trap. **d** The photonic laser interrogates the trapped ion 0.4 Hz quadrupole clock transition and is disciplined every 20 ms using optical frequency correction feedback. Interleaved with the clock discipline cycle is a 20 ms qubit SPAM experiment operation cycle. **e** Clock transition stabilization and qubit generation scheme using coil-stabilized 674 nm SBS laser to directly drive the 5^2^S_1/2_ to 4^2^D_5/2_ optical clock transition. Energy levels of the strontium trapped ion showing the optical clock transition at 674 nm (*S*_1*/*2_ → *D*_5*/*2_) which has a natural linewidth of 0.4 Hz. Laser pulses probe the left and right sides of the 0.4 Hz quadrupole transition sequentially. Externally applied cooling and state detection lasers and sequencing are also shown. Clock stability is measured by comparing two ^88^Sr^+^ optical clocks and measuring the difference (Δ). **f** Qubit operation and SPAM scheme using Zeeman splitting to create a coherent qubit with the 5^2^S_1/2_ transition. Optical pumping with the coil-stabilized 674 nm SBS laser from the upper 5^2^S_1/2_ qubit state to the 4^2^D_5/2_ shelving state, then qubit quench to the 5^2^P_3/2_ state, and then spontaneous emission to the lower qubit 5^2^S_1/2_ state are shown. Optical pulse sequencing for coil-stabilized 674 nm SBS laser state preparation and qubit probe. External laser pulse sequencing for qubit cooling/quench, optical pumping, and state detection. ECDL - external cavity diode laser. Si_3_N_4 -_ silicon nitride. SBS - Stimulated Brillouin Scattering. PDH - Pound-Drever-Hall. SEIT - Surface electrode ion trap. SPAM - state preparation and measurement.

There has been success towards miniaturizing the trapped ion physics package^6–8^ and integrating functions into the surface electrode trap (SET)^9^, including detection^10–12^, control electronics^13^ and optical beam delivery^14–16^. However, two of the most limiting optical subsystems in terms of size, power, complexity, and reliability, are the low phase noise clock interrogation laser and the ultra-low expansion optical cavity used to stabilize this laser^17^ which can comprise more than half the experiment volume^18^. These systems are used to mitigate laser frequency noise and instability which play an important role in performance in qubit systems^15,19^. Today, these requirements are fulfilled by meter-scale environmentally isolated cavity stabilized external cavity diode lasers (ECDLs)^20^ tailored to each precision atomic transition wavelength (Fig. 1a) to reduce the laser integral linewidth (ILW), fractional frequency noise (FFN), and drift. In addition to the linewidth, reduction in laser high frequency noise is becoming increasingly important as the speed of qubit control and logic operations are increased and as the performance of precision quantum applications demand lower crosstalk^21–23^. The Brillouin laser is an important approach to mitigate high frequency noise. Short-wave IR fiber Brillouin lasers have been used to interrogate the strontium trapped ion optical clock transition^24^. However, such approaches are bulky and require power inefficient nonlinear frequency conversion. Moving away from fiber implementations, miniaturized low noise lasers^25^ and stabilization cavities^26^ have achieved lab-scale domain performance, but are not readily integrated on-chip. There has been progress towards integrated low noise lasers and cavities in the ultra-low loss silicon nitride (Si_3_N_4_) integration platform^27–32^. However, demonstration of record chip-scale laser performance in room temperature trapped-ion experiments capable of performing trapped-ion qubit operations^1^ including SPAM, Rabi oscillations and Ramsey interferometry, has remained elusive.

Here, we report record chip-scale stabilized laser performance with an integrated Brillouin laser stabilized to a 3-meter long coil resonator cavity in a room temperature trapped-ion experiment. We then utilize this laser to perform optical clock interrogation and qubit SPAM operations in a room-temperature ion trap without a bulk-optic ultra-low expansion (ULE) reference cavity or optical frequency conversion. We characterize the coil-stabilized laser stability through the self-comparison of interleaved optical clocks on the ^88^Sr^+^ ion transition and characterize the linewidth through high-resolution trapped ion spectroscopy. The coil-stabilized SBS laser achieves an ADEV of 8.8 × 10^−13^ at 20 ms which then enables interrogation of the ^88^Sr^+^ 0.4 Hz quadrupole optical clock transition. Disciplining the laser to the ion via optical clock protocols further stabilizes the laser and results in a short-term instability averaging down as 5.3 $\times {10}^{-13}/\sqrt{\tau }$, and a 1.5 kHz ion-stabilized laser linewidth. The combination of the nonlinear high-frequency noise reduction of the Brillouin laser with the mid-frequency noise reduction provided by the low thermo-refractive noise (TRN) limit^30,32^ of the 3-meter coil resonator (quality factor (Q) of 93 million) results in a 14 Hz FLW and 322 Hz reverse 1/π ILW. We also quantify the relative detuning of the disciplined laser from the ion transition in its local time reference frame through the self-comparison of two interleaved clock feedback loops, measuring a root mean square (rms) frequency deviation of 180 Hz relative to the ion local reference frame over a 100 s measurement period. To perform qubit operations, we interleave alternating shots of qubit experiments with clock interrogation sequences, enabling us to robustly perform trapped ion qubit operations such as spectroscopy, Rabi oscillations and Ramsey interference, as well as demonstrate qubit SPAM with 99.6% fidelity. These results demonstrate a clear path for the CMOS foundry compatible Si_3_N_4_ photonic platform to realize stabilized visible light lasers and other optical components integrated with the trapped ion surface trap, enabling chip-scale quantum compute and optical clock solutions.

## Results

### Integrated photonic coil resonator stabilized Brillouin laser

The experiment utilizes a chip-scale photonic laser, which consists of a silicon nitride (Si_3_N_4_) Brillouin (SBS) laser and Si_3_N_4_ coil-resonator reference, and a room-temperature strontium ^88^Sr^+^ surface ion trap (SET) chip (left to right Fig. 1c). Both the SBS laser and 3-meter coil resonator operate directly at 674 nm without wavelength conversion and replace the traditional meter-scale ULE cavity and table-top fiber Brillouin lasers (Fig. 1a) used in prior cryogenic trapped-ion demonstrations^24^. We discipline the photonic laser Fig. 1d (left to right) to the optical clock transition of a strontium ^88^Sr^+^ ion trapped on a room temperature SET with an optical clock protocol, making a small frequency correction each cycle with an acousto-optic modulator (AOM). To perform qubit experiments, we alternate between clock cycles which discipline the laser to ^88^Sr^+^ clock transition and qubit cycles (every 20 ms). This allows us to perform a series of qubit experiments including qubit SPAM. Details of the clock interrogation protocol and qubit operation protocol, including atomic transitions, cycles, lasers, and timing, are summarized in Fig. 1e and  1f, respectively. In future work, to facilitate further integration, the feedback signal can be applied directly to the laser for frequency correction removing the need for the AOM^33^. The photonic laser and other lasers and passive and active components can be integrated with the platform compatible SET in the future to realize an integrated trapped-ion quantum experiment as illustrated in Fig. 1b.

**Fig. 2:**
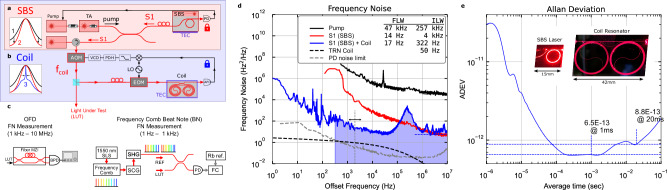
Photonic integrated ultra-narrow linewidth stabilized 674 nm Brillouin laser. **a** The 674 nm SBS laser reduces the pump fundamental linewidth and high frequency noise, leading to reduction in linewidth wing energy (1→ 2 in Fig. 2a). The pump laser is an ECDL which is amplified prior to the SBS laser using a 674 nm tapered amplifier. **b** The SBS laser is locked using an AOM frequency shifter to the quadrature point of the integrated 3-meter coil resonator to reduce the close to carrier noise and the integral linewidth (blue line 3 in Fig. 2b) using an EOM. The output of the SBS laser is boosted using a 674 nm injection locked laser prior to delivery to the trapped ion clock transition. **c** The frequency noise over the range of 1 Hz to 10 MHz of the coil-stabilized SBS laser is measured over two ranges using two techniques. An unbalanced fiber MZI OFD for the 1 kHz to 10 MHz range (left side). For the 1 Hz to 1 kHz range, the frequency noise is measured with a frequency counter on a beatnote between the coil-stabilized SBS laser and a 1 Hz ULE cavity stabilized fiber frequency comb converted to 674 nm using SCG and SHG. **d** Frequency noise plots for the pump (black), SBS S1 tone (red), and the coil stabilized SBS laser (blue). Traces from the OFD and comb beatnote measurements are stitched together at 1 kHz. Also shown is the coil TRN floor (dashed black curve) and the PD noise limit (dashed gray curve). The peak in the SI + coil (blue) curve is the PDH lock servo bump. FLW for the pump, SBS unstabilized output and SBS stabilized output are measured to be 47 kHz, 14 Hz and 17 Hz respectively, showing a high frequency noise reduction of a factor of over 3 orders of magnitude. 1/π reverse ILW for the pump, SBS unstabilized output and SBS stabilized output are measured to be 257 kHz, 4 kHz and 322 Hz respectively, showing a reduction of almost 3 orders of magnitude. The TRN floor predicts that the ILW can be reduced to 50 Hz with feedback loop lock improvements. **e** The ADEV of the coil stabilized SBS laser showing stability of 6.5 ×10^−^^13^ at 1 ms and 8.8 × 10^−^^13^ at 20 ms. Photographs of operational SBS laser and coil resonator shown in inset. SBS - Stimulated Brillouin Scattering. AOM - Acousto-optic modulator. MZI - Mach-Zehnder Interferometer. OFD - Optical frequency discriminator. FSR - Free spectral range. FLW - Fundamental linewidth. ILW – Integral linewidth. TRN - Thermorefractive noise. SN - Shot noise. ECDL – External cavity diode laser. EOM – Electrooptic modulator. S1 – SBS first order Stokes Tone. PD – Photodiode. ADEV – Allan Deviation. SCG – Super continuum generation. SHG – Second harmonic generation.

The experimental coil stabilized laser is shown in Fig. 2a and 2b. In addition to the SBS laser and coil resonator chips, the laser system consists of a 674 nm ECDL laser that serves as the SBS pump laser, which is boosted by a tapered amplifier (TA). The light is then amplified by an injection locked laser and sent to the trapped ion (further details in Methods). In the future, the pump laser can be replaced with a silicon nitride platform compatible extended cavity tunable laser (ECTL)^34,35^. The level of amplification can be reduced through reduction of losses by integrating all components on-chip and adding needed amplification through on-chip integrated gain^36^. Details of the coil resonator design are given in Supplementary Note 2. The pump laser is locked to the Si_3_N_4_ SBS resonator using a proportional-integral-derivative (PID) lock loop (details in Supplemental Note 1). The SBS process provides nonlinear noise suppression of the high frequency noise^37^ (lineshapes 1→ 2 in Fig. 2a). The SBS laser is then locked to the coil resonator using an acousto-optic modulator (AOM) frequency shifter resulting in integral linewidth reduction (lineshapes 2→ 3 in Fig. 2b) using an electro-optic modulator (EOM). In the future, the AOM can be replaced by directly driving the Brillouin laser frequency using a thermal or piezo-electric actuator^33^.

The coil resonator serves as an intermediate holdover cavity to reduce the FFN^30,31^ for interrogating the ion clock transition and its short term stability is matched to the 20 ms SPAM and qubit cycle. The 3-meter length provides a large mode volume that lowers the thermo-refractive noise (TRN) floor and provides a sharp discriminant slope for PDH lock^32^. The coil resonator has a propagation loss of 0.66 dB/m, 93 million intrinsic quality factor (*Q*_*i*_), and a loaded quality factor (*Q*_*l*_) of 54 million (see Supplementary Note 2). The coil has a free spectral range of 65.5 MHz with multiple discrimination peaks available within the AOM 200 MHz tuning range enabling robust Pound-Drever-Hall (PDH) locking.

The frequency noise is measured using two techniques to cover the full 1 Hz to 10 MHz range (described in further detail in the Methods Section). For 1 kHz to 10 MHz, we use an unbalanced Mach-Zehnder Interferometer (MZI) optical frequency discriminator (OFD)^32^ (left side of Fig. 2c). For the 1 Hz to 1 kHz range, we measure the beatnote of the laser with an ultra-low expansion (ULE) cavity stabilized fiber frequency comb that is second harmonic generation (SHG) frequency doubled to 674 nm and use a frequency counter to measure the frequency noise and to calculate the ADEV. Frequency noise measurements from 1 Hz to 10 MHz and linewidth calculations are shown in Fig. 2d for the free running pump laser (black), the SBS laser S1 output without coil resonator lock (red), and the SBS laser locked to the coil resonator (blue). The SBS laser 14 Hz FLW is a reduction of several orders of magnitude from the 257 kHz free-running pump FLW and the coil stabilized SBS laser reverse 1/π ILW is 322 Hz, a reduction of an order of magnitude from the 4 kHz 1/π ILW of the unstabilized SBS laser. The slight increase in high frequency noise for the coil-locked SBS is due to the width of the servo lock bump and additive noise from the electronics, and the lower frequency noise features from 30 Hz to 100 Hz are environmental noise for the packaged coil. The TRN limited noise floor provides a lower limit on performance that can be achieved with this coil (Fig. 2d black dashed curve), yielding a TRN limited ILW of *<* 50 Hz. This limit can be reached with a further optimized PDH lock loop and improved coil packaging.

The Allan Deviation (ADEV) for the coil locked SBS is shown in Fig. 2e along with photographs of the operational SBS laser and coil-resonator (inset of Fig. 2e). The ADEV of an unpackaged coil stabilized SBS laser is calculated from the full 1 Hz to 10 MHz frequency noise spectrum and shows a minimum of 6.5 × 10^−13^ at 1 ms and 8.8 × 10^−13^ at 20 ms. Data showing the ADEV measured with MZI OFD for the pump, coil locked pump, and the coil locked SBS are shown in Supplementary Fig. 3b. However, this data is only valid for frequencies above 10 kHz since the fiber MZI is not stabilized and drifts for longer time scales, as seen in the upturning of all ADEV curves in SI Fig. 3b. Further details of the ADEV and cavity stabilized comb measurements are given in Supplementary Notes 5 and 8, respectively. The coil resonator is packaged with epoxied optical fibers and placed inside a temperature stabilized enclosure (details in Supplementary Note 4).

**Fig. 3:**
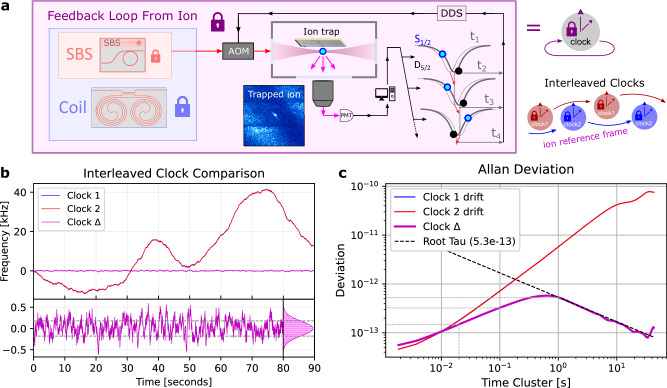
Coil and SBS laser stabilization using a trapped ion. **a** The integrated coil stabilized SBS laser described in Fig. 2 is further stabilized to the optical clock transition of the trapped ion qubit using an adapted clock protocol to interrogate the S_1/2_ and D_5/2_ transition at the FWHM points and apply a correction to the AOM frequency during the next loop cycle. **b** Running two interleaved clock feedback loops to the ion optical clock transition in parallel allows us to compare their performance (by taking the frequency difference) and verify robust locking as each lock loop (red and blue) shows the same underlying drift of the photonic coil resonator in their applied correction. The difference of the two locks (purple) shows the relative frequency stability of the laser after disciplining the laser to the ion optical clock transition. The RMS deviation of this frequency difference shows the loops are bounded within 180 Hz of each other (dashed lines), estimating the frequency stability that would be seen by an interleaved qubit. **c** Fractional frequency instability (Allan Deviation) of the frequency difference of the two clocks shows a short-term instability averaging down as $5.3\times {10}^{-13}/\sqrt{\tau }$. SBS - Stimulated Brillouin Scattering. AOM - Acousto-optic modulator. FWHM – Full width at half maximum. RMS – Root mean square.

### Characterization of coil resonator using the trapped ion

We characterize the performance of the coil stabilized Brillouin laser using the trapped ion 0.4 Hz linewidth quadrupole transition (*S*_1*/*2_ ↔ *D*_5*/*2_) to measure laser noise at timescales longer than those possible with the OFD. The ion is trapped using an in-house fabricated surface electrode trap within a compact room temperature ultra-high-vacuum (UHV) chamber (see Supplementary Note 3). In addition to the 674 nm SBS laser, we employ 422 nm, 1092 nm, and 1033 nm non-integrated lasers for the cooling, quench, and state detection cycles, respectively (Fig. 1b). In the future, these lasers can also be integrated using the platform compatible silicon nitride ECTL laser design. Broad spectroscopy scans performed with the stabilized laser clearly show transitions between the *S*_1*/*2_ and *D*_5*/*2_ sub-levels, which are Zeeman split by a 5.9 Gauss magnetic field to avoid frequency crosstalk with nearby electronic and motional transitions. To reduce the effect of drift that could skew our spectroscopy data, we utilize a “waterfall” approach instead of a sequential scanning approach that would measure each frequency many times before proceeding to the next frequency. With the waterfall approach, we acquire data at a single sample per frequency at a time and then loop over the frequencies many times. The probability of transition at each frequency is displayed in real-time as the statistics are acquired, giving the appearance of a ‘waterfall’ of spectral features. This spectroscopy approach ensures the linewidth can only be broadened by residual drift and not artificially narrowed. To measure the coarse stability of the thermally stabilized photonic coil resonator we repeatedly perform trapped-ion spectroscopy scans on the coil-stabilized ECDL and record the linewidth and center frequency. We measure a typical coil drift over the course of an hour to be less than 100 kHz. For these measurements, the coil resonator is packaged as described in Supplementary Note 4. To estimate the dependence of drift rate on temperature, we track the coil drift after changing the temperature setpoint by 1 mK. We observe a coil frequency shift of ~ 2.5 MHz in half an hour (1/e settling time of 7 min). This suggests that our observed frequency stability of 100 kHz over an hour is equivalent to a temperature stability of 30 µK.

We track the drift of the coil and simultaneously calibrate the frequency for qubit operations using the optical clock transition and a frequency calibration protocol similar to those used for optical clocks (Fig. 3a) which we implement via the software control ARTIQ^38^. As illustrated in Fig. 3a, we probe the left side of the ion transition at the full-width-half-max (FWHM) of the laser limited linewidth and record if the ion is bright or dark after fluorescence detection (2 ms) to indicate if the ion was excited to the D state by the laser pulse or not. We repeat this process on the other side of the resonance at FWHM and record if the ion was bright or dark. If the laser is on resonance, both samples will have equal probabilities of excitation. If the laser has drifted, then the side to which the laser has drifted will be excited to the D state with higher probability. To correct laser drift, the software feedback loop applies a small optical frequency correction, typically about 40 Hz, in the next cycle. This feedback loop maintains balance between the excitation probabilities on each side of the ion resonance, disciplining the laser to the local ion clock transition. The duration of our clock interrogation protocol is limited by the detection time (2 ms) and cooling time (2 ms) for the ion between cycles. The ion probe linewidth, ion transition sampling points, and ion reference frame lock stability are illustrated in Fig. 3a. This protocol not only disciplines the laser to the ion, it also allows us to precisely measure the laser drift relative to the ion. We also test the resilience of the lock to the ion by adding a triangle wave frequency ramp to the final AOM while clocking, which introduces an artificial *drift* for which the calibration protocol must cancel out. Further, we run two interleaved feedback loops in parallel, with only one having a triangle wave and one without, verifying the feedback loop accurately recovers the input triangle wave from the difference of the two and that the lock is robust to artificial drift rates of more than 4 kHz/s.

To characterize the laser stability, we run two interleaved ion clock interrogation feedback loops (Fig. 3b) and compare their relative (difference) frequency^38^. Figure 3b shows that the center carrier frequency remains within 180 Hz RMS of each other, which is smaller than the linewidth of 1.8 kHz used to probe the ion (Fig. 3b). 180 Hz RMS corresponds to a fractional stability for the SBS + coil + ion of 4.1×10^-13^ in the trapped ion reference time frame. Further details of longer term coil drift and clock interrogation are given in Supplementary Note 7.

We calculate the Allan deviation of the relative stability of the interleaved loops to the ion reference time frame in Fig. 3c, which shows that the short-term instability averages down as $5.4\times {10}^{-13}/\sqrt{\tau }$. It is important to note that the ion clock transition itself may have shifts and instabilities from various sources such as blackbody radiation (BBR), and the laser will inherit any such instability of the ion. In future work, we plan on comparing the ion disciplined laser relative to a precision reference to measure absolute stability more precisely. However, for reliable qubit operations disciplining to the ion (including any systematic shifts) is desired as the clock protocol therefore continuously calibrates the laser frequency to control the qubit despite any absolute instability of the ion frequency.

Currently, the same ion is used for both clock interrogation and qubit operations, which take turns interleaving single shots with the ion, which is reset and re-initialized each shot. In the future, this overhead could be reduced through dedicated trapped-ion clock calibration zones on a fully integrated system on a chip (Fig. 1f). The self-comparison interleaved technique we use to measure the short term frequency stability relative to the local ion time reference frame has been widely studied^38–40^ in the context of optical clocks. It is important to note that we do not measure absolute stability as that would require comparison to an optical clock, MASER, or a cavity stabilized frequency comb, which we plan to do in future work that explores optical clock applications.

### Qubit state preparation and measurement (SPAM)

**Fig. 4:**
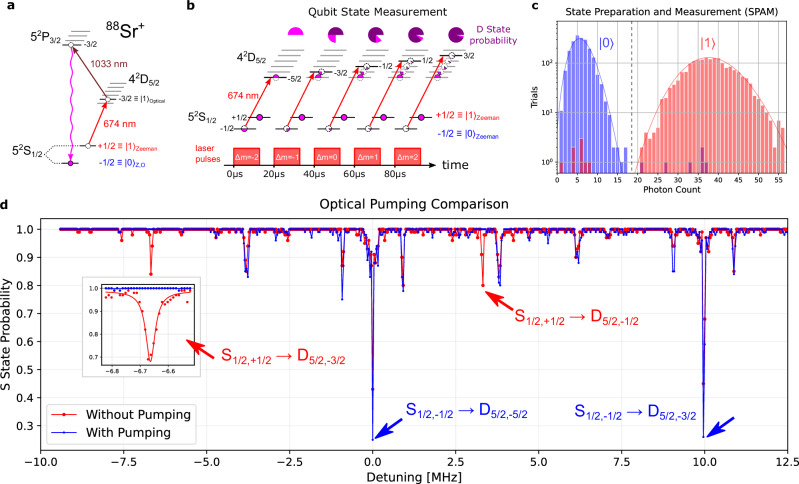
Qubit operations for state preparation and measurement using photonic coil stabilized laser. **a** Energy level diagram of frequency selective optical pumping scheme. First a 674 nm pulse excites any population in the *S*_1*/*2,+1*/*2_ state to the *D*_5*/*2,−3*/*2_ state, then a 1033 nm pulse pumps population to the *P*_3*/*2,−3*/*2_ state, from which it decays to the *S*_1*/*2,−1*/*2_ state. Each cycle of this sequence increases the probability the ion is in the *S*_1*/*2,−1*/*2_ state. **b** For high fidelity state detection of a Zeeman qubit, one of the ground state spins must be shelved in the *D*_5*/*2_ state before fluorescence detection. A single pulse of the 674 nm laser is capable of shelving most of the population but is limited by the coherence. Therefore, we apply pulses to all available D state sub-levels to ensure a high probability of shelving the desired Zeeman sub-level of the S state into the metastable D state. **c** Histogram of photon counts over 2000 trials showing a total SPAM fidelity of 99.6%. **d** Ion spectroscopy with (blue) and without (red) optical pumping, shows that spectral lines initiating from the *S*_1*/*2,+1*/*2_ states vanish with optical pumping. Optical pumping is performed on the *S*_1*/*2,+1*/*2_ → *D*_5*/*2,−3*/*2_ (at laser detuning f = −6.67 MHz) and the *S*_1*/*2,−1*/*2_ → *D*_5*/*2,−5*/*2_ (f = 0 MHz) is used for qubit operations. The *S*_1*/*2,+1*/*2_ → *D*_5*/*2,−1*/*2_ (f = 3 MHz) transition also vanishes with optical pumping and the *S*_1*/*2,−1*/*2_ → *D*_5*/*2,−3*/*2_ (f = 10 MHz) remains. Inset: a close up of the *S*_1*/*2,+1*/*2_ → *D*_5*/*2,−3*/*2_ shows over 99% state preparation fidelity from the ratio of the amplitudes with and without optical pumping using only the coil + ion stabilized laser. SPAM - state preparation and measurement.

Qubit SPAM for quantum computation^41^ is typically achieved using frequency selective narrow linewidth optical clock transitions. To perform operations such as Rabi oscillations and ion spectroscopy, we interleave two cycles continuously, a feedback loop cycle of the clock interrogation protocol which disciplines the laser to the ion and a qubit measurement cycle. This allows us to reliably address the Zeeman split trapped-ion qubit as shown in the ^88^Sr^+^ energy diagram in upper right of Fig. 4a and perform qubit SPAM (Fig. 4b and 4c). We then use qubit calibration measurements (e.g., Ramsey interferometry) to characterize the ion disciplined laser coherence. First, we characterize the performance of only the pump ECDL locked to the 674 nm integrated coil resonator and disciplined to the ion. We then characterize the performance of the coil stabilized SBS laser disciplined to the ion. In both cases we perform full qubit SPAM of a room temperature ^88^Sr^+^ trapped ion, achieving over 99% fidelity, with the full coil stabilized SBS laser we measure 99.6% SPAM fidelity (Fig. 4c).

The experimental procedure for interleaving clock feedback with qubit operations is illustrated in Fig. 1d. First, we interrogate the 0.4 Hz quadrupole clock transition of the ^88^Sr^+^ ion clock transition to discipline the coil stabilized laser through frequency correction as described above. Next, the laser is used to drive the trapped ion to prepare, probe and measure qubits, while the coil resonator acts as a holdover cavity for the laser. This process repeats as necessary to acquire the statistics needed for qubit measurements (typically 100 trials per qubit scan datapoint) to measure fidelities and coherence time. Because we characterize the relative stability of two interleaved clock measurements of the ion, we can use this to predict that the interleaved qubit will experience the same relative stability. Over the course of 100 clock interrogations the feedback loop stabilizes to a time independent frequency deviation relative to the ion. Therefore, over the duration of many qubit experiments the qubit will experience frequency detuning less than 180 Hz.

For qubit state preparation (see Fig. 4a), we initialize the qubit in the *S*_1*/*2,−1*/*2_ state via frequency selective optical pumping out of the *S*_1*/*2,+1*/*2_ state. First, we pulse the 674 nm laser on the *S*_1*/*2,+1*/*2_ → *D*_5*/*2,−3*/*2_ transition for 15 *µs*. Next, we pulse a 1033 nm laser for 50 *µs* to excite population from *D*_5*/*2,−3*/*2_ to *P*_3*/*2,−3*/*2_, which then rapidly decays to the *S*_1*/*2,−1*/*2_ state. We then repeat this pulse sequence ten times. We next characterize the performance of the ultra-low phase noise coil stabilized SBS laser in trapped ion wide scan spectroscopy. Figure 4d shows two spectroscopy scans, with the blue trace taken with optical pumping operations and the red trace without optical pumping operations. Both traces in Fig. 4d employ the SBS + coil + ion laser. Conclusive evidence of state preparation can be seen in the absence of the *S*_1*/*2,+1*/*2_ → *D*_5*/*2_ transitions in the spectroscopy with optical pumping operations. The spectroscopy scans also verify state preparation by showing that the depth of the *S*_1*/*2,−1*/*2_ → *D*_5*/*2_ transitions has increased. The high resolution of the spectroscopy scans taken with the SBS laser resolves all peaks with high SNR, demonstrating one benefit of the low linewidth wing energy for the SBS laser. State preparation fidelity *>* 99% is confirmed in the inset of Fig. 4d which shows the amplitude of the *S*_1*/*2,+1*/*2_ → *D*_5*/*2,−3*/*2_ transition with and without optical pumping, performed with just the ion disciplined coil stabilized ECDL.

For qubit state measurement we use the coil and ion stabilized laser to shelve one of the S state sub-levels into the D state (Fig. 4b). First, we apply a 15 *µs* pulse on the *S*_1*/*2,−1*/*2_ → *D*_5*/*2,−5*/*2_ transition to shelve most of the *S*_1*/*2,−1*/*2_. However, the fidelity of a single pulse is limited by the finite laser coherence time of the laser. Therefore, we apply additional pulses to shelve the same S state sub-level into the other available D state sub-levels. Once we have shelved the *S*_1*/*2,−1*/*2_ into the D states, we apply resonant 422 nm light on the *S* → *P* transition, along with 1092 nm light to repump the ion during cycling, and count photons with a photo multiplier tube (PMT). Performing this procedure over 2000 trials for preparation and detection of each state we measure a total SPAM fidelity of 99.6%, (histograms in Fig. 4c). We next compare fidelity with and without the SBS laser. With the coil stabilized Brillouin laser disciplined to the room temperature trapped ion, we observe that only two pulses are necessary to achieve *>* 99% fidelity (Fig. 4c) whereas with just the coil + ion ECDL laser, we require 5 pulses to achieve over *>* 99%. This improvement in time and efficiency is due to the increased coherence time provided by the SBS laser, which makes each pulse more efficient in transferring population from the *S*_1*/*2,−1*/*2_ to the *D*_5*/*2_ sub-levels.

### Qubit sensing for measurement of ion-coil stabilized laser coherence

**Fig. 5:**
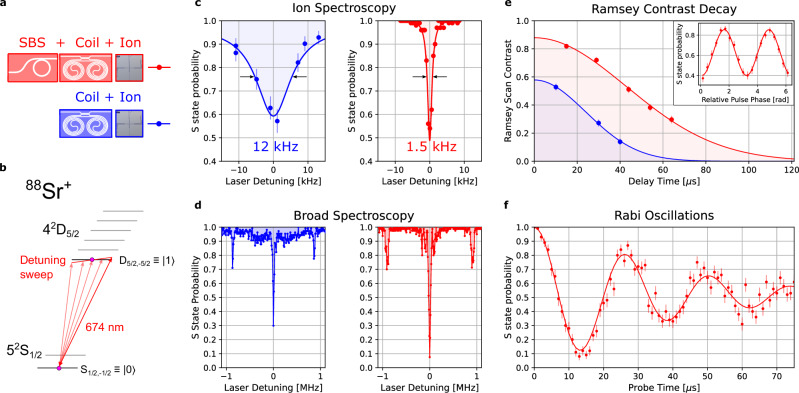
Coherent qubit operations with a photonic and ion stabilized laser source. **a** Comparison of the qubit operations and coherence of the coil stabilized pump laser while clocked to the ion (coil + ion, blue) and the coil stabilized SBS laser while clocked to the ion (SBS + coil + ion, red). **b** Energy level diagram of the strontium trapped ion optical qubit. **c** Trapped ion spectroscopy with the coil + ion stabilized pump laser (blue) showing a linewidth of 12 kHz, while the SBS + coil + ion stabilized laser (red) shows a linewidth of 1.5 kHz. Error bars indicate the standard error of the proportion of S state probabilities. **d** Wider spectrum showing the motional sidebands of the trapped ion near ±900 kHz. The SBS laser (red) suppresses the servo bumps that are otherwise seen in the coil + ion stabilized spectroscopy (blue). **e** Ramsey contrast vs. delay time measuring the laser noise of the coil stabilized laser (blue, 30 *µs*) and the SBS laser stabilized to coil (red 60 *µs*). Inset, shows the data for a single delay time as the phase between the two pulses is varied. Error bars indicate the standard error of the Ramsey contrast mean. **f** Rabi oscillations with the SBS + coil + ion laser. Error bars indicate the standard error of the proportion of S state probabilities. SBS - Stimulated Brillouin Scattering.

The quadrupole transition of ^88^Sr^+^ has been previously used for quantum computation^5^ and here we use the qubit to quantify the laser performance and ability to perform SPAM. We find that when the table-top ECDL is stabilized to the coil and ion stabilized (lower blue in Fig. 5a) the resulting coherence is not adequate for reliable qubit operations due to the high frequency offset noise (Fig.5d). With the noise reduction provided by the stabilized SBS laser, and reduced optical cross talk between transitions, we are able to probe the qubit at the longer pulse times required for qubit operations. To highlight the improved performance, we perform waterfall spectroscopy and measure a 1.5 kHz linewidth for the coil stabilized Brillouin laser disciplined to the trapped ion. In contrast, with just the coil stabilized ECDL disciplined to the ion (without the Brillion laser), we measure a 12 kHz linewidth (Fig. 5c).

To measure the coherence of the lasers with the qubit we perform Ramsey interferometry on the trapped ion by applying two $\frac{\pi }{2}$ pulses to the ion (*S*_1*/*2_ ↔ *D*_5*/*2_) while sweeping the relative phase of the pulses. This ensures we capture the maximum contrast at each delay time (Fig. 5e, inset). We then fit the decay of contrast of these Ramsey fringes as the delay time between the two $\frac{\pi }{2}$ pulses is increased. For the disciplined coil stabilized ECDL laser alone, we observe a 1*/e* decay time of 33 *µs* and for the SBS + coil + ion stabilized laser we observe 60.5 *µs* (Fig. 5e). We next compare ion spectroscopy using the disciplined SBS coil stabilized laser to just the disciplined coil stabilized ECDL laser (Fig. 5d), illustrating the performance when the servo bumps are removed for free running measurement (250 kHz). To quantify the fidelity of single qubit rotations we perform Rabi oscillations on the trapped ion optical clock transition (*S*_1*/*2_ ↔ *D*_5*/*2_) using the trapped-ion disciplined coil stabilized SBS laser (Fig. 5f). We first prepare the ion with Doppler cooling (Fig. 1f), optical pumping (Fig. 4a,d), and then pulse the 674 nm laser for varying pulse lengths. With the ion disciplined coil-stabilized SBS laser we observe single flip fidelity of 92%, which is limited by the laser coherence time. This is in contrast to the single flip fidelity achieved with only the ion disciplined coil-stabilized ECDL that yields an 80% contrast due to the shorter coherence time.

These qubit experiments use clock interrogation probe points and pulse parameters designed to match the laser limited linewidth to give the tightest feedback to discipline the laser to the ion. However, the drift of the coil can cause the laser to lose lock from the probe window of $\pm 0.7$ kHz relative to the ion. The laser can quickly be recaught and relocked to the ion so that qubit experiments can resume, but it is impractical to have frequent re-locks during qubit experiments. With the narrow probe clock parameters ($\pm 0.7$ kHz) the lock is robust enough to perform 100 s of qubit cycles over many seconds. At longer time scales the coil drift makes it difficult to remain disciplined to the ion using the +/− 0.7 kHz frequency sampling offset points. If a continuous lock is required, for instance measuring the averaging down time over 1000 s of seconds, we increase the probe sampling points from $\pm 0.7$ kHz to $\pm 25$ kHz to ensure the laser does not drift out of the probe frequency offset window, enabling the lock to be continuously maintained for hours (Supplementary Figs. 5, 6 and 7). The practical tradeoff in increasing the probe points from $\pm 0.7$ kHz to $\pm 25$ kHz loosens the feedback loop such that the relative frequency detuning of the disciplined laser increases from 180 Hz to 1 kHz relative to the ion time reference frame. However, this is still more than sufficient for qubit state preparation and measurement and smaller than the laser limited linewidth of 1.5 kHz.

## Discussion

In this work, we report a significant advance in photonic chip-scale stabilized visible light lasers for room temperature trapped-ion experiments. We demonstrate record laser stability, frequency noise, and linewidth with an integrated 674 nm Brillouin laser locked to an integrated 674 nm 3-meter long coil resonator that is disciplined to the 0.4 Hz quadrupole optical clock transition for a ^88^Sr^+^ ion trapped in an integrated room temperature SET based experiment. The SET is compatible with silicon nitride monolithic integration of the laser and other silicon nitride components that have been demonstrated to date, providing a clear path towards full trapped ion system integration as illustrated in Fig. 1b. Importantly, these results are achieved by directly driving the strontium ultra-narrow atomic clock transition at a visible wavelength without wavelength conversion, reducing the needed integration complexity and improving the potential laser to ion transition power delivery efficiency. Using this high-performance laser, we demonstrate full qubit operations including trapped-ion spectroscopy, qubit SPAM, Rabi oscillations, and Ramsey coherence measurements, without use of a bulk-optic tabletop reference cavity or second harmonic generation. Further, the reduced high frequency offset noise delivered by the SBS clock laser provides very high SNR, widely tunable, trapped ion spectroscopy and increased qubit coherence time compared to using the coil stabilized ECDL pump laser alone. Note added in proof: During the review process of this manuscript, we became aware of related work using a silicon nitride coil to stabilize a lab-scale second harmonic generation converted 1348 nm laser to interrogate the optical clock transition of a cryogenic trapped strontium ion^42^.

While the coherence of the laser sets a limit to the efficiency of each optical pumping pulse, it does not limit the reliability or fidelity of optical pumping, as the pulse sequences can be repeated many times, with each further depleting the unwanted spin population. After 20 rounds of optical pumping pulses, the state preparation fidelity exceeds our measurement fidelity. Figure 4d shows through the absence of excitation events from the ${S}_{1/2,-1/2}\to {D}_{5/2,-3/2}$ and ${S}_{1/2,-1/2}\to {D}_{5/2,-1/2}$ that there are no instances of the ion being excited from the ${S}_{1/2,-1/2}$ state within the 100 trials per shot of each data point near optical transitions, consistent with 100% state preparation fidelity. However, the clock laser coherence can limit the SPAM fidelity if it is sufficiently poor, as the efficiency of each shelving pulse is set by the coherence of the laser relative to the pulse time. Similar to how the Rabi oscillations in Fig. 5f show about 92% inversion efficiency at 13*μs*, each shelving pulse will have a finite efficiency. Because the other D state transitions are weaker, they are slower and can have efficiencies of 75%, still limited by the coherence time. After five shelving pulses this is sufficient to shelve the ion in a D state 99.9% of the time. A more fundamental limit to the detection fidelity is set by the finite lifetime of the metastable D state, as decays cause errors. More sophisticated detection schemes could be employed in the future to overcome this limit. Further, we show that with the ion disciplined coil stabilized SBS laser we observe significantly reduced background high frequency noise (Fig.5d) on top of the longer coherence times from the narrower fundamental linewidth and ultra-low high frequency phase noise. Whereas, while the coil stabilized ECDL alone can be disciplined to the ion and perform SPAM, our measurements show limited utility for long coherent qubit pulses due to significant background noise (Fig.5d) that will create crosstalk to nearby transitions.

The potential for this chip-scale stabilized laser design to reach lower noise and linewidth is determined in part by the current resonator TRN floor, shown in Fig. 2e (black dashed curve), which predicts an achievable ILW of less than 50 Hz through designs enhancements that can reduce the high frequency noise and fundamental linewidth^43–45^. In terms of linewidth and frequency noise, there are multiple time regimes of importance for trapped ion experiments. The fundamental 14 Hz linewidth is relevant to faster time scale operations less than order 1 μs. The 322 Hz ILW includes carrier jitter and is a measure of the Voight broadened lineshape which is relevant to the experimental timescales and operations between approximately 1 μs and the 20 ms interrogation time. To further reduce the ILW of the laser, next steps include implementing a large mode volume coil SBS design^46^, improving the feedback servos and optimizing their bandwidths, utilizing a lower noise silicon nitride integrated pump ECTL laser^34,47^. We estimate an order of magnitude reduction in pump ILW, which has been demonstrated for the silicon nitride ECTLs, or in frequency noise by the SBS laser, which has been demonstrated using a coil Brillouin laser design^46^, can achieve a TRN limited ILW of ~ 300 Hz for the SBS laser on its own.

Further, advancement in photonic resonator stabilization performance such as using different materials to reduce drift^48^ suggests that there is room for improvement in the visible wavelengths which could enable even narrower visible light linewidths and lower phase noise, a subject of current ongoing research. Furthermore, development of heterogeneously integrated laser pump sources in the visible^29,49,50^ would enable locating the pump laser on chip and a fully integrated chip-scale photonic-ion platform.

For position, navigation and timing (PNT) applications, such as clocking, the stability of the lasers have significant room for improvement. For example, we observe that intensity stability of the loaded integrated coil-resonator is a key source of drift that while improved with active intensity stabilization, is still susceptible to polarization instability which maps into intensity stability via the TM mode of the integrated coil resonator. Polarization stabilization on an integrated chip^51^ to the coil is one approach that can readily reduce the drift by an order of magnitude and longer coil-resonator^52^ can further reduce the TRN and in addition to specialized packaging can further improve the longer term stability. In future work, we plan to address this issue to achieve better performance over long averaging times and improve stability.

The results reported here are critical and direct steps towards monolithic integration within a trapped ion quantum processor. The path towards integration of a trapped-ion quantum system, such as an optical clock, as illustrated in Fig. 1b, can build on prior work in the ultra-low loss Si_3_N_4_ platform^27^ which is a CMOS foundry compatible platform that itself is compatible with ion trap chip. For example, the ion trap surface electrodes can be fabricated directly on top of the silicon nitride photonic layers^15^, with openings in the trap electrode metal, through which silicon nitride grating couplers compatible with all required wavelengths can emit and focus light to the ion trapped above the surface. The 674 nm SBS pump and other lasers required for trapped ion qubit operation can be implemented using the same Extended Cavity Tunable Laser (ECTL) designs with the appropriate semiconductor gain block^34,35^. An important benefit of the silicon nitride ECTL design, is the immunity to optical feedback and isolator free operation^34^. Direct laser frequency and cavity control can be utilized to remove the need for bulky, power consuming AOM frequency shifters^53^. Such laser and resonator control can be achieved using thermal or piezoelectric PZT actuators, that operate across the visible and NIR, directly integrated on the silicon nitride platform^33,54^. Delivering light to the trapped ion through grating beam emitters^15,16^ is compatible with all integrated laser and optical components. Directly locating all components on the same chip can remove phase noise that accumulates in free space and fiber connected experiments and reduces the required laser output power. The optical power to the ion can be increased using ECTL dual gain chip designs and further amplification of the Brillouin light can be achieved using heterogeneous semiconductor amplifier and injection locked amplification.

Miniaturization through integration can enable the creation of arrays of portable quantum information processors for computation and sensing. Multiplexing the ion clocks on chip as well as multiplexing the laser stabilization devices on chip could create a path to much tighter phase stability clock protocols among multiple trapped-ions which interleave varying interrogations across various witness qubits and scaling the number of trapped ions on chip into arrays, allowing local entanglement operations on chip. Additionally, ultra-low noise chip-scale lasers can be stabilized and locked to atomic and qubit transitions providing compact techniques for pre-stabilization of the world’s most stable lasers and highest precision atomic sensing experiments^55^. These approaches could enable robust and deployable quantum technologies, with applications for inertial navigation, quantum sensing, quantum computing, and space-based quantum applications.

## Methods

### Clock transition laser system

The SBS laser starts with a 674 nm pump laser which is a commercial external cavity diode laser (MOGLabs Littrow cavity laser) which provides 18 mW of optical power to seed a commercial tapered amplifier (MOGLabs TA). The TA amplifies the optical power of the seed laser to 200 mW using 700 mA of TA current. The excess optical power available makes it possible to use a 50:50 fiber splitter to send light to the SBS chip and split off the back reflected S1 light from the SBS. 100 mW of power exits the splitter and is sent to the SBS chip. We estimate -6dB input and output coupling loss to the SBS chip, such that 25 mW of power enters the SBS on chip, which is more than enough to saturate S1. The SBS light (S1) returns through the 50:50 splitter, after which there is 1 mW of optical power which enters an optical circulator, sending 400uW of SBS light to an injection locked laser for amplification to 150 mW.

The pump laser is locked to the SBS resonator using the MOGlabs inbuilt proportional-integral-differential (PID) servo in the controller of the laser. The controller also modulates the current at 250 kHz to add sidebands for the lock. The laser comes with a 700 kHz photodiode for locking and the SBS resonator transmission is detected using this photodiode. Demodulation is carried out internally in the controller and a current servo is added with the drive current for the lock. This Pound-Drever-Hall (PDH) lock is a ‘weak’ lock, i.e., it keeps the pump laser on resonance with the SBS resonator but does not provide any linewidth reduction.

SBS lock to coil: After the optical circulator 70 mW of injection lock amplified SBS light is sent to a double pass AOM which acts as the servo to lock the SBS to the coil. 5 mW is sent to the coil and 15 mW is sent to another double pass AOM, after which 1 mW is delivered post fiber to the ion. For locking SBS laser to the coil, an avalanche photodetector (Thorlabs APD430A) is used for detecting transmission. A voltage controlled oscillator (VCO) drives a resonant electro optic modulator (EOM) at 25 MHz to add sidebands for PDH lock. Demodulation is carried out by mixing the APD signal with the power tapped from VCO to generate the error signal. A commercial low noise PIID servo (Vescent D2-125) is used to provide feedback to a double pass acousto-optic modulator (AOM) for the frequency control by adding the servo signal to the VCO controlling the AOM. See also Supplementary Note 1: Details of lock.

### Trapped ion surface electrode trap and physics package

The surface electrode ion trap chip was designed and fabricated in the UMass Amherst clean room facilities. The surface electrodes are composed of a 1.1 µm thick layer of sputtered niobium metal, deposited onto a 4 in. fused silica wafer. The electrodes are defined via reactive ion etching (RIE) which transfers the optical lithography pattern to the niobium. The wafers are then diced into 1 cm square chips and cleaned with argon ion milling, which removes ~ 100 nm of niobium and anneals the surface. See also Supplementary Note 3: Ion trap fabrication.

### Laser frequency noise and ADEV measurements

The laser frequency noise is measured using two different techniques to cover the 1 Hz to 10 MHz Frequency range. For 1 kHz and above, an optical frequency discrimination (OFD) method using a fiber Mach-Zehnder Interferometer (MZI) is used. For the 1 Hz to 1 kHz range, the beat note (BN) between the laser under test and a ultra-low expansion (SLS cavity) stabilized frequency comb (Vescent Photonics) that has been converted to the visible range with second harmonic generation (SHG) is measured using a frequency counter. The unbalanced fiber MZI free-spectral range (FSR) frequency is calibrated with a radio frequency (RF) source by transmitting the laser through the MZI and ramping the phase shift on one arm to get peak to peak voltage of the photodetector output. With the laser under test input to the calibrated MZI OFD the output power change corresponds to an optical frequency shift around the quadrature point. The MZI detector output is trigged on an oscilloscope around the quadrature point and sampled at different rates for different offset ranges. The data is averaged down over several measurements and stitched together for different sampling rate traces to obtain the full range frequency noise data. The OFD measurement is limited by fiber MZI environmental noise that occurs below 1 kHz offset. For the beat note noise measurement between the laser under test and the ULE stabilized C-band frequency comb the 100 MHz repetition rate comb output is frequency-doubled to generate a 674 nm comb line. The beat note signal is measured with a frequency counter and the inherent stability of the SLS allows us to measure frequency noise in offsets of 1–2000 Hz. Due to the limited optical power of the comb and the nonlinear efficiency of the frequency doubling, at 674 nm, we phase lock the beat note signal to a Voltage-Controlled Oscillator (VCO) to improve the signal-to-noise ratio for the frequency counter. The frequency noise from the beat note measurement and the MZI OFD measurements are stitched together at 2 kHz offset and converted. Final frequency noise data sets are converted to Allan-Deviation (ADEV) data using post processing.

## Supplementary information

Supplementary Information

Transparent Peer Review file

## Data Availability

The data that support the plots within this paper and other finding of this study are available from the corresponding author upon request with specific reasons why data is needed and conformation with ethical and legal requirements.

## Code availability

The codes that support the findings of this study are available from the corresponding authors upon request with specific reasons why data is needed and conformation with ethical and legal requierments.
