# Supplementary material for: Chip scale coil stabilized Brillouin laser driving a room temperature trapped ion qubit
Source: Nat Commun. 2026 Mar 3;17:3982. doi: 10.1038/s41467-026-69948-2 (PMC13135068; doi:10.1038/s41467-026-69948-2)
Supplement: Supplementary file 1 — Supplementary Information [file 41467_2026_69948_MOESM1_ESM.pdf]

Supplementary Information

Chip Scale Coil Stabilized Brillouin Laser Driving a Room Temperature Trapped Ion Qubit

Nitesh Chauhan[1*], Christopher Caron[2*], Andrei Isichenko[1], Meiting Song[1],
Zhenyu Wei[2], Nishat Helaly[2], Kaikai Liu[1], Jiawei Wang[1], Robert J. Niffenegger[2†], Daniel J. Blumenthal[1†]

[1]*Department of Electrical and Computer Engineering, University of California Santa Barbara, Santa Barbara, CA 93106*
[2]*Department of Electrical and Computer Engineering, University of Massachusetts Amherst, Amherst, MA 01003*
Corresponding Authors: †rniffenegger@umass.edu, danb@ucsb.edu
**These authors contributed equally*

(Dated: February 24, 2024)

In this Supplementary Information, we outline more details on the locking setups, design of the SBS and coil resonator and the enclosure to thermally stabilize the resonators.

Supplementary Note 1: Details of lock

SBS Lock: The 674 nm pump laser is a commercial external cavity diode laser (MOGLabs Littrow cavity laser) which provides 18mW of optical power to seed a commercial tapered amplifier (MOGLabs TA). The TA amplifies the optical power of the seed laser to 200mW using 700mA of TA current. The excess optical power available makes it possible to use a 50:50 fiber splitter to send light to the SBS chip and split off the back reflected S1 light from the SBS. 100mW of power exits the splitter and is sent to the SBS chip. We estimate -6dB input and output coupling loss to the SBS chip, such that 25mW of power enters the SBS on chip, which is more than enough to saturate S1. The SBS light (S1) returns through the 50:50 splitter, after which there is 1mW of optical power which enters an optical circulator, sending 400uW of SBS light to an injection locked laser for amplification to 150mW.

The pump laser is locked to the SBS resonator using the MOGlabs inbuilt proportional-integral-differential (PID) servo in the controller of the laser. The controller also modulates the current at 250 kHz to add sidebands for the lock. The laser comes with a 700 kHz photodiode for locking and the SBS resonator transmission is detected using this photodiode. Demodulation is carried out internally in the controller and a current servo is added with the drive current for the lock. This Pound-Drever-Hall (PDH) lock is a 'weak' lock, i.e. it keeps the pump laser on resonance with the SBS resonator but does not provide any linewidth reduction.

SBS lock to coil: After the optical circulator 70mW of injection lock amplified SBS light is sent to a double pass AOM which acts as the servo to lock the SBS to the coil. 5mW is sent to the coil and 15mW is sent to another double pass AOM, after which 1mW is delivered post fiber to the ion. For locking SBS laser to the coil, an avalanche photodetector (Thorlabs APD430A) is used for detecting transmission. A voltage controlled oscillator (VCO) drives a resonant electro optic modulator (EOM) at 25 MHz to add sidebands for PDH lock. Demodulation is carried out by mixing the APD signal with the power tapped from VCO to generate the error signal. A commercial low noise PIID servo (Vescent D2-125) is used to

provide feedback to a double pass acousto optic modulator (AOM) for the frequency control by adding the servo signal to the VCO controlling the AOM.

Supplementary Note 2: Resonator design

The resonators are based on low loss dilute mode Si_3N_4 waveguides with a waveguide width of 2.3 μm and core thickness of 40 nm. The core is made of stoichiometric Si_3N_4 deposited with low pressure chemical vapor deposition process, the lower cladding is 15 μm thermal oxide and the upper cladding is 6 μm, formed with plasma enhanced chemical vapor deposition[1]. The waveguide supports the fundamental TE (TE0) and fundamental TM (TM0) modes, and the cross section is shown in Supplementary Figure 1a. The resonators are designed in the lower loss TM0 mode which also has a larger mode area of 2.4 μm^2 compared to the TE mode area of 1.4 μm^2. The critical bend radius, defined as radius above which bend loss contribution is < 0.01 dB/m, is ~ 3.5 mm for the TM0 mode. This waveguide design is used for SBS resonator and for the bus and coupling section of the coil resonator. The coil section of the coil resonator has waveguide width of 3.25 μm to reduce the bend losses and further increase the area of the mode to 3.1 μm^2. The resonator coupling for both the coil and the SBS resonator is designed to only support the TM0 mode with critical coupling. The coil is processed in a 200 mm CMOS process. The Q and FSR of the coil obtained by calibrated MZI method[1,2]. The measured Q_l = 54 million, Q_i = 93.2 million with a propagation loss of 0.66 dB/m and FSR of 65.5 MHz is obtained, shown in Supplementary Figure 1b, which is the lowest waveguide loss and highest Q at 674 nm. The SBS resonator is designed so that both the pump and ~25 GHz redshifted first Stokes tone (= Brillouin gain shift) are both resonant in the resonator[3], and the FSR of 8.33 GHz is chosen such that 3x FSR = Brillouin gain shift. The SBS resonators are fabricated in a 100 mm wafer in university cleanroom and demonstrates higher loss likely due to higher bend loss contribution resulting from fabrication variations increasing the critical bend radius, with propagation loss of 1.6 dB/m, Q_l of 16 $\times 10^6$ and Q_i of 39.6×10^6, shown in Supplementary Figure 1c. The SBS resonator demonstrates a threshold of 10 mW (on chip power) and a 40 mW S2 threshold. The SBS resonators are operated just below S2 threshold for lowest fundamental linewidth operation[2,3]. Future designs can improve the threshold by fabricating the CMOS devices also in the CMOS process.

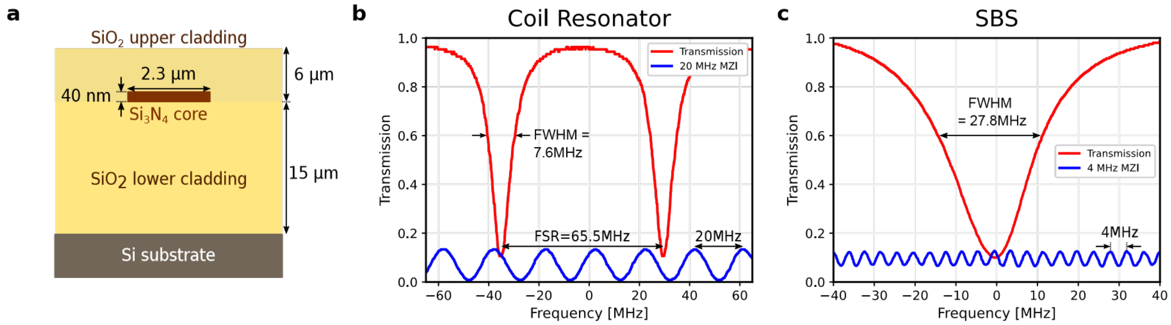

Supplementary Figure 1. Resonator design and characterization: (a) waveguide cross section. **(b)** Q and FSR measurements of the coil resonator with Q_l of 54×10^6, Q_i of 93.2×10^6 propagation loss of 0.66 dB/m and a measured FSR of 65.5 MHz. **(c)** Q and FSR measurements of the SBS resonator with Q_l of 16×10^6, Q_i of 39.6×10^6 propagation loss of 1.6 dB/m.

Supplementary Note 3: Ion trap fabrication

The surface electrode ion trap chip was designed and fabricated in the UMass Amherst clean room facilities. The surface electrodes are composed of a 1.1 μm thick layer of sputtered niobium metal, deposited onto a 4 in. fused silica wafer. The electrodes are defined via reactive ion etching (RIE) which transfers the optical lithography pattern to the niobium. The wafers are then diced into 1 cm square chips and cleaned with argon ion milling, which removes $\sim$ 100 nm of niobium and anneals the surface.

Supplementary Note 4: PIC packaging

Temperature stabilized aluminum enclosures (Supplementary Figure 2b) house the integrated coil to isolate it from the environment. Cleaved fibers are aligned and epoxied to the facet of the coil, which is then placed upon Teflon (Supplementary Figure 2c) inside the inner aluminum enclosure. The inner enclosure is mounted on a TEC (Supplementary Figure 2b) and stabilized by a temperature controller (Vescent Slice-QTC) to provide thermal control within 1 mK. This aluminum box is placed inside an additional aluminum box and then a Styrofoam box for further isolation. The SBS device is not packaged, but is temperature stabilized. The device is coupled with cleaved fibers using nano-positioners (Thorlabs Nanomax) and index matching gel is used to reduce facet loss. The facet loss for the packaged coil is 5.5 dB/facet and is mostly expected from the misalignment during the packaging process, the facet loss for the SBS resonator is $\sim$ 2 dB/facet.

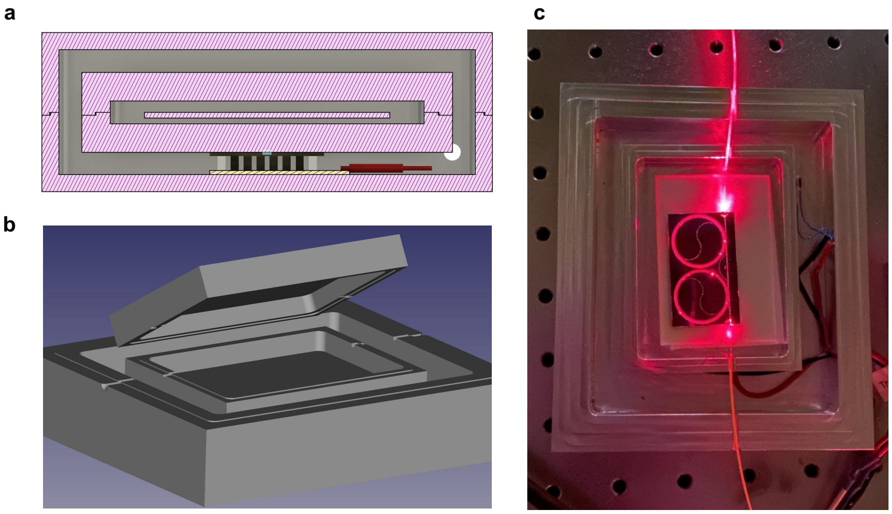

Supplementary Figure 2. **Enclosure: (a)** Side view of the dual aluminum enclosures for the packaged coil with a TEC in between. **(b)** 3D view of the enclosures. **(c)** Packaged coil on top of Teflon sheet (white) within enclosure.

Supplementary Note 5: Laser Frequency noise and Allan Deviation

The laser frequency noise data from the main text Figure 3 (repeated below in Supplementary Fig. 3a) can be used to calculate an Allan deviation to estimate the fractional stability over various time scales. Calculating the ADEV from the data in Supplementary Fig. 3a leads to the solid curves in Supplementary Fig. 3b, which are measurements using the OFD for offset frequency noise of 1 kHz and greater. The coil TRN frequency and ADEV floors are shown in both plots as dashed black curves. The OFD frequency noise and ADEV plots are not valid for offset frequencies greater than 10 kHz due to OFD fiber environmental fluctuations, and can be seen in the premature upturning ADEV solid curves to the left of the vertical dashed line in Supplementary Fig. 3b (the OFD measurement region). At longer times, the ADEV of the pump locked to an unpackaged coil was measured using a Stable Laser System (SLS) reference cavity stabilized 100 MHz repetition rate fiber optic frequency comb that is frequency doubled down to 600 nm (see Supplementary Note 6). The comb and SLS cavity are further stabilized to a rubidium clock that is provisioned to GPS. Frequency noise data was taken down to 1 second by heterodyne beating the coil stabilized laser and measuring with a frequency counter. The resulting ADEV is shown by the dashed grey curve to the right of the vertical dashed line in Supplemental Fig. 3b, indicating an ADEV of approximately 3.5E-12 at 20 ms for the unpackaged coil. The coil was then packaged as described above in Supplementary Note 4 in the ion trapping lab. We expect that the packaged coil performance improves the ADEV to match the OFC solid blue curve and this verification is the subject of future work. The performance of the coil as a holdover cavity in this work supports an order of magnitude improvement from packaging.

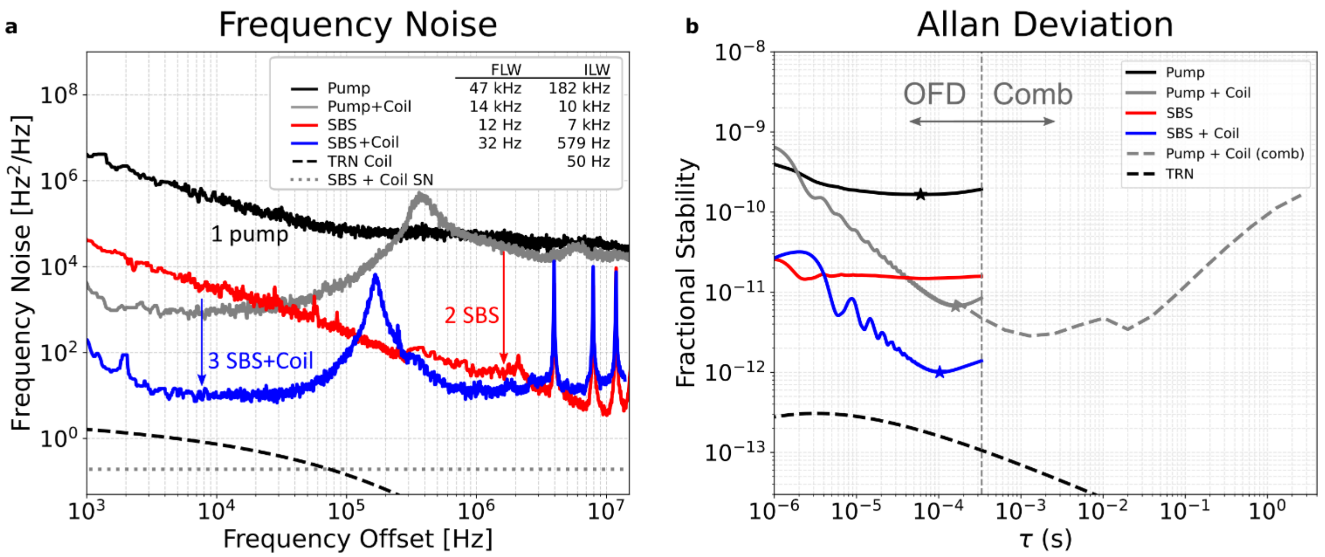

Supplementary Figure 3. **Laser Frequency Noise and Allan deviation: (a)** Laser frequency noise of various laser and stabilization stages including pump, SBS, and coil stabilized SBS as measured by the MZI OFD (also shown in Figure 3 in the main text). **(b)** Allan deviation (ADEV) of the data in (a) is shown in solid curves to left of vertical dashed line. Unpackaged coil ADEV as measured with 1 Hz optical frequency comb (grey dashed curve). See Supplemental Note 6 for further details on the comb beatnote stability measurement.

Supplementary Note 6: Stabilized Optical Frequency Comb Measurement of Unpackaged Coil Resonator

The frequency noise is measured with a combination of the fiber MZI optical frequency discriminator (OFD) and a heterodyne beat-note with cavity-stabilized fiber frequency comb. The frequency comb is self-referenced and the optical refence frequency is locked to a Hz-level ULE-cavity-stabilized C-band laser. The comb is disciplined to a rubidium frequency standard referenced to a GPS receiver. The stabilized comb light is frequency doubled to 674 nm, combined with the coil-stabilized laser using a 50:50 fiber coupler, and detected on a photodetector. The beat-note signal is electronically filtered to isolate an individual comb line and recorded on a frequency counter. The OFD provides short-term frequency noise at > 1 kHz offset frequencies, while the comb beat-note is used for < 1 kHz offsets long-term drift characterization.

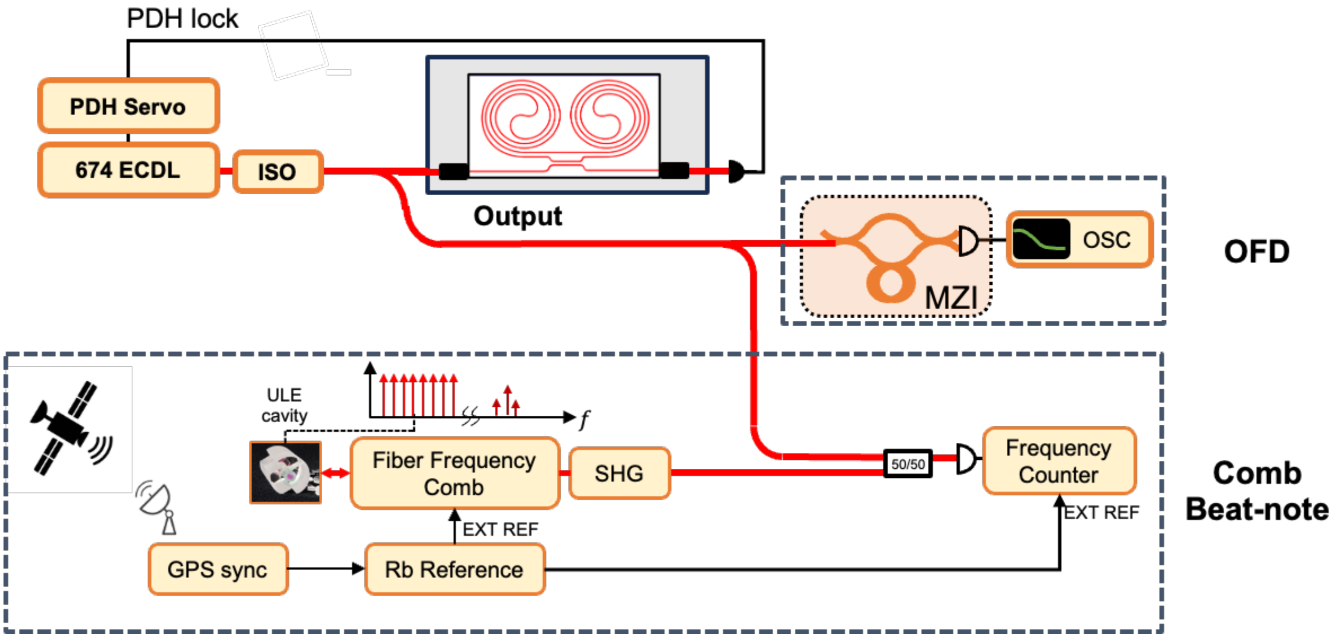

Supplementary Figure 4. Stabilized optical frequency comb beatnote measurement of unpackaged coil resonator stability: 1 Hz cavity stabilized optical frequency comb disciplined to a GPS stabilized rubidium atomic clock. The comb is wavelength converted after super continuum generation, to provide a reference tooth at 674 nm to measure the coil lock stability for the unpackaged coil. The 674 nm ECDL laser is PDH locked to the coil reference cavity and the output is beat note detected with the stabilized comb. A frequency counter is used to measure the carrier stability. This method is used to quantify frequency noise from 1 Hz to about 1 kHz and stitched together with frequency noise data in the 1 kHz to 10s of MHz range made with a fiber MZI OFD. The beatnote measurements are used to calculate the ADEV for the unpackaged device as shown in Supplemental Figure 3b.

Supplementary Note 7: Longer term coil drift, clock interrogation, and interleaved clocks

Day-to-day over the course of many months we find that the resonance of the coil does not drift far away from the ion. Measuring the coil stability via spectroscopy (not clocking) we have observed that over the course of 160 minutes that the coil deviates by 400 kHz after an hour but returns to its original frequency (Supplementary Figure 5a) an hour later. In practice this means that we usually find the same ion clock

transition, closer to where we left it than any other possible transition between different sublevels of the S and D states, the nearest of which is 2.4MHz away.

If there are abrupt thermal changes to the room (such as a power outage which resets the thermal enclosure control), we do observe faster drift. However, even the dual clocking algorithm can track this faster drift continuously for over 40 minutes (Supplementary Fig. 3b).

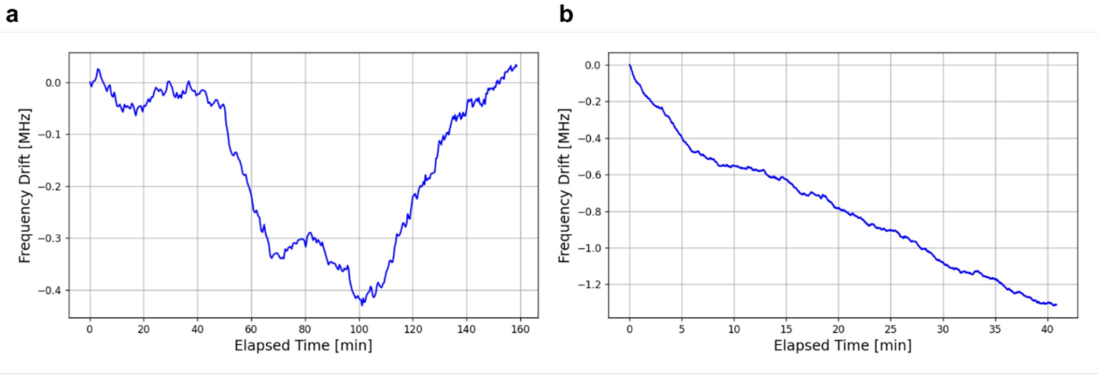

Supplementary Figure 5. **Coil Drift:** (a) Coil drift measured over the course of 160 minutes. (b) Coil drift measured over the course of half an hour while the trapped ion clocking is continuously tracking the laser drift.

Tracking deviations between the two clock locks during this 40-minute period allows us to calculate an averaged down Allan Deviation (ADEV). We observe that the fractional frequency stability of dual interleaved clocks average down as $5\times 10^{-12}/\sqrt{\tau}$ (Supplementary Fig. 6 left side), this underestimates the stability that would be seen by a single clock, which would be $1/\sqrt{2}$ faster and average down at $3.5\times 10^{-12}/\sqrt{\tau}$. This allows the clock to reach a stability of 1.1×10^{-13} at 1,000 seconds, during the 40 minutes (2,500 seconds) of continuous clocking shown in Supplementary Fig. 6 left side. We believe that drift from the thermal fluctuations of the coil induced by intensity and polarization fluctuations limit this stability and that future improvements could readily allow the ADEV to average down better than $1\times 10^{-13}/\sqrt{\tau}$. Narrowing the probe pulse frequency offset improves the measured ADEV at the expense of reliable lock limited duration of 20 seconds (middle plot Supplementary Fig. 6). Comparison of the two regimes and measured ADEV as limited by the probe pulse offset and tradeoffs are summarized in right plot in Supplementary Fig. 6.

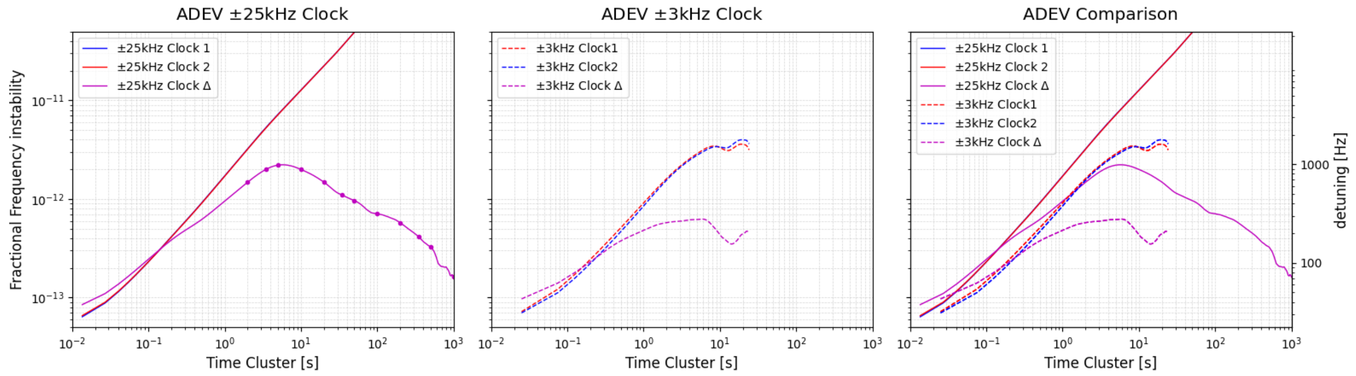

Supplementary Figure 6. **Fractional Frequency stability over 1,000 seconds:** Power broadening of the probe offset frequency from +/- 25 kHz offsets with a shorter pulse time (see top of Supplemental Fig. 7) allows sampling to keep up with drift over longer time periods and measurements out to 1000 seconds (left plot). This increase in

sampling offset frequency allows longer operation with the tradeoff in net linewidth or stability. Tracking the deviations of the interleaved dual clocks in SI Figure 4b allows us to calculate the Allan Deviation as it averages down for longer clock experiments up to 1,000 seconds. Fitting the slope of the dual clocks gives $5 \times 10^{-12}/\sqrt{\tau}$, with the performance of a single clock estimated to be $1/\sqrt{2}$ better and averaging down at $3.5 \times 10^{-12}/\sqrt{\tau}$, such that a single clock would reach a stability of 1.1×10^{-13} at 1,000 seconds. Compared to the data with +/- 3 kHz frequency offset yields a higher stability from 20 ms to 10 seconds, but the data does not have time to average down due to the limited due to lock stability at longer time scales for this probe frequency offset.

We compare the different scenarios for disciplining the laser for optimized short term stability and for longer term stability as summarized in Supplementary Fig. 7. Pulse diagrams for the two scenarios are shown in the top of Supplementary Fig. 7 right and left sides for +/- 3 kHz offset and +/- 25 kHz offset respectively. For the best short term fractional frequency stability, it is beneficial to use the narrowest probe frequency offset to interrogate the ion optical clock transition (left column of Supplementary Fig. 7). For the current experimental system that is 6kHz. However, with such a narrow probe, there is a chance that the coil drift can cause the laser to become unlocked due to dynamic range of loop components, for example maximum frequency detuning of the AOM. For qubit applications this does not pose issues, as the laser can be quickly relocked in software and qubit preparation can proceed. However, for optical clock type measurements such as time averaging down over 1000s of seconds (right column Supplementary Fig. 7), it is necessary for the lock to remain engaged for as long as possible. To accomplish this, we can artificially power broaden the laser probe linewidth and a shorter laser pulse time. As an example, we show data (Supplemental Fig. 7 (right column) with a +/- 25 kHz frequency offset that is able to reliably discipline the laser to the ion for over 40 minutes while still maintaining relative frequency comparable to the narrowest linewidth probe lock.

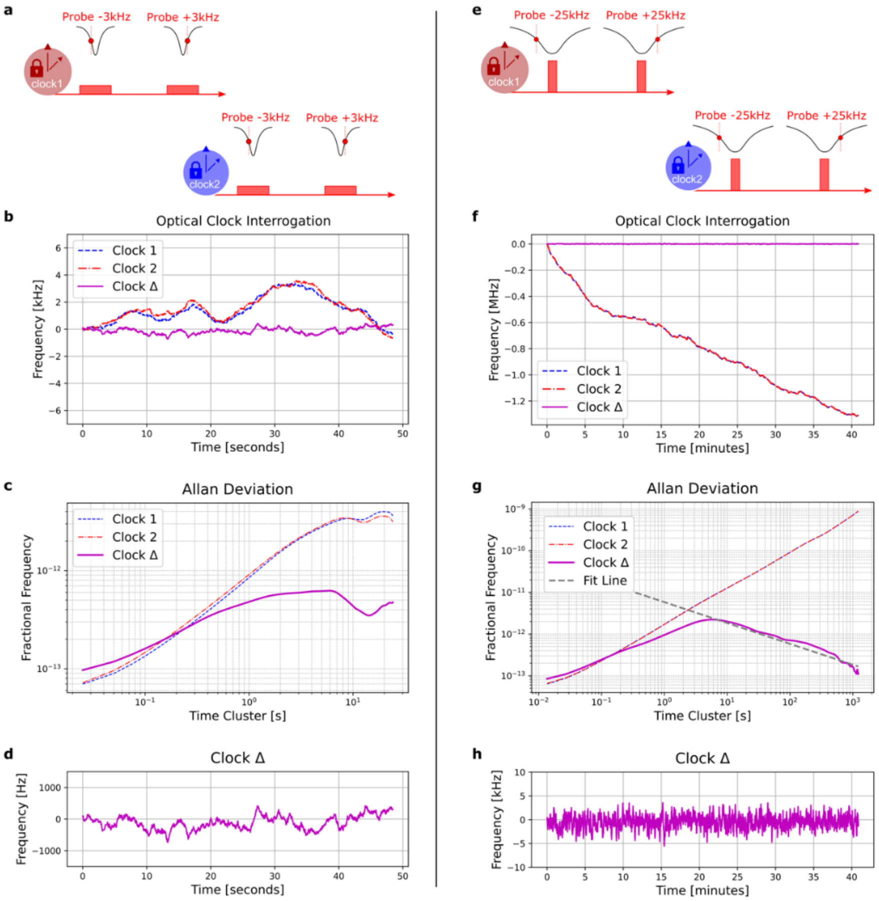

Supplementary Figure 7. **Clock probe linewidth and lock tightness:** Left panels, laser limited linewidth (6kHz) probe of the optical clock transition for disciplining the laser. Right panels, artificially power broadened linewidth probing of the ion for feedback and disciplining to the ion that is more robust to drift of the coil.

Supplementary References

1. Chauhan, N. *et al.* Ultra-low loss visible light waveguides for integrated atomic, molecular, and quantum photonics. *Opt. Express, OE* **30**, 6960–6969 (2022).
2. Gundavarapu, S. Sub-Hz Fundamental Linewidth Silicon Nitride Integrated Brillouin Lasers and Their Applications. (UC Santa Barbara, 2018).
3. Behunin, R. O., Otterstrom, N. T., Rakich, P. T., Gundavarapu, S. & Blumenthal, D. J. Fundamental noise dynamics in cascaded-order Brillouin lasers. *Phys. Rev. A* **98**, 023832 (2018).
